# Y-chromosomal haplotyping of single sperm cells isolated from semen mixtures – a successful identification of three perpetrators in a multi-suspect sexual assault case

**DOI:** 10.3325/cmj.2014.55.537

**Published:** 2014-10

**Authors:** Lei Feng, Cheng Xu, Xianhai Zeng, Huaicai Zhang, Fan Yang, Wanshui Li, Zheng Tu, Caixia Li, Lan Hu

**Affiliations:** 1 Institute of Forensic Science, Ministry of Public Security, Beijing, People’s Republic of China; 2 Key Laboratory of Forensic Genetics, Ministry of Public Security, Beijing, People’s Republic of China; 3 Chinese People’s Public Security University, Beijing, People’s Republic of China; 4 Forensic Technology Division, Public Security Bureau of Fujian Province, Fuzhou, People’s Republic of China; 5 Institute of Criminal Science and Technology, Public Security Bureau of Hangzhou City, Hangzhou, People’s Republic of China

## Abstract

**Aim:**

To obtain individual Y-short tandem repeat (STR) profiles in a multi-suspect sexual assault case.

**Methods:**

We used laser cut microdissection to capture the single sperm cell in the multi-contributor semen sample, combined with the low volume polymerase chain reaction (LV-PCR) method to genotype the single sperm cell profiles using the Yfiler® kit. Consensus DNA profiles were generated from 5 replicate experiments.

**Results:**

Ninety-four parallel LV-PCRs were performed and 41 reactions (44%) produced Y-STR profiles with more than nine loci. Three individual Y-STR profiles were successfully obtained.

**Conclusion:**

The three Y haplotype units matched three known perpetrators’ genotypes. Our results showed that single sperm cells Y-STR analysis was a powerful method for analyzing multi-donor semen mixture sample.

Multi-suspect sexual assault is a crime frequently encountered by forensic scientists. Current standard procedures, including preferential lysis, are incapable to separate the sperm DNA of different donors. In this way, a mixture profile is often obtained, which can only be used for exclusion rather than identification. Another method to isolate single cells from mixtures that has been successfully used in the forensic community is laser cut microdissection (LCM) (1-5). However, by means of this method it is still challenging to get the autosome short tandem repeat (STR) profile for semen mixtures with more than two contributors. This is because of the random assortment of chromosomes in meiosis (6). An alternative strategy to analyze male DNA is Y-STR analysis. In our laboratory, we previously established LCM system and low volume polymerase chain reaction (LV-PCR) platform for biological mixture analysis (7). Here, we developed a method of single sperm cells Y-STR analysis combining LCM and LV-PCR, which was successfully used in a sexual assault case.

## Case background

In May 2012, a drunken woman was sexually assaulted in a hotel room and a video recording indicated three men as suspects. No other evidence but a vaginal swab was collected from the victim. Using preferential lysis method to separate the sperm cells, the sperm DNA was purified by a commercial kit. We got a mixed DNA profile of more than two contributors, by which it was difficult to exclude or identify suspects. The victim’s vaginal swab was the key evidence, so we re-analyzed this sample by LCM platform to genotype the perpetrators’ DNA for forensic analysis. The analysis was focused on genotyping the Y-STR of single sperm cells.

## Materials and methods

### Sample collection

A single-source semen sample was collected on tissue paper from one healthy volunteer, who had given informed consent. Three perpetrators’ semen samples were also collected on tissue paper. The victim’s vaginal swab had been collected previously by local police. All the samples were air dried overnight and stored at room temperature (25°C) until needed.

### Routine DNA detection

Standard “in-tube” DNA amplification was performed to verify the result of single sperm assay. The single source semen sample and three perpetrators’ sperm samples were treated with MagAttract^®^ DNA Mini M48 kit (Qiagen, Hilden, Germany) to extract genome DNA according to the manufacturer’s guidelines. The equivalent of 1 ng DNA was amplified using the AmpFlSTRs Y filer^®^ kit (Applied Biosystems, Foster City, CA, USA).

The case swab sample was treated with preferential lysis method to separate sperm cells and epithelial cells. The sperm cells DNA was extracted with MagAttract® DNA Mini M48 kit and 1 ng DNA was amplified with AmpFlSTRs Y filer^®^ kit.

### Single sperm separation with LCM

*Step 1 – slide preparation.* The tissue paper with volunteer’s semen (0.5 cm^2^) or swab sperm specimens were placed in 500 µL ddH_2_O and incubated for 60 minutes at 37°C in a shaking metal bath. After centrifugation and removal of the supernatant, the cell pellets were resuspended in 30 µL ddH_2_O and smeared onto a UV-sterilized polyethylene naphthalate membrane slide (Carl Zeiss Ltd, Jena, Germany). The slide was air dried at room temperature.

*Step 2 – sperm isolation and lysis.* Sperm isolation was performed with a PALM MicroBeam instrument (Carl Zeiss Ltd) as reported previously (8). Each sperm cell was captured onto one AG480F AmpliGrid®slide reaction site (Advalytix AG, Munich, Germany). Ninety-two assays were performed for single source sample and 94 assays for case sperm samples.

For cell lysis, 0.75 µL lysis buffer (0.1 mg/mL proteinase K, 4 mM DTT) was added to each reaction site and sealed with 5 µL mineral oil (Advalytix AG). Cells were lysed at 56°C for 2 hours and boiled for 10 minutes on an AmpliSpeed Cycler (Advalytix AG).

### PCR and electrophoresis

LV-PCR was performed with AG480F AmpliGrid slide on AmpliSpeed Cycler. The PCR mixture contained 3.7 µL PCR Reaction Mix, 2.0 µL Primer Mix, 0.2 µL 25 mM MgCl_2_, and 4 U AmpliTaq Gold DNA Polymerase. An aliquot of the mixture (0.75 µL) was added to each reaction site after cell lysis. Control DNA 9947A (Applied Biosystems, 0.1 ng/mL) was used as positive control, and no DNA template was used as negative control. PCR conditions were as follows: preincubation at 95°C for 15 minutes; 34 cycles of denaturation at 94°C for 1 minute, annealing at 61°C for 1.25 minutes, and extension at 72°C for 1.25 minutes; followed by a final elongation step at 60°C for 1 hour. For the single source semen sample and three perpetrators’ sperm samples, PCR was amplified according to the AmpFlSTRs Y filer^®^ kit guidelines.

All the PCR products (including 1.5 µL PCR product and 5 µL sealing oil) were denatured in 10 µL loading buffer, which was composed of HI-DI^TM^ formamide and LIZ^TM^-500 size standard mixture (Applied Biosystems) in a proportion of 500:1 (volume in volume). For the single source semen sample and three perpetrators’ sperm samples, 1 μL amplified DNA was denatured in 10 μL of loading buffer. DNA was separated by capillary electrophoresis using an ABI 3500xl Genetic Analyzer (Applied Biosystems). The peak detection threshold was set at 50 RFU, and the data analysis was performed using GeneMapper^TM^ ID X (Applied Biosystems) software.

## Results

### DNA profiling of single source semen specimens

**Figure 1:**
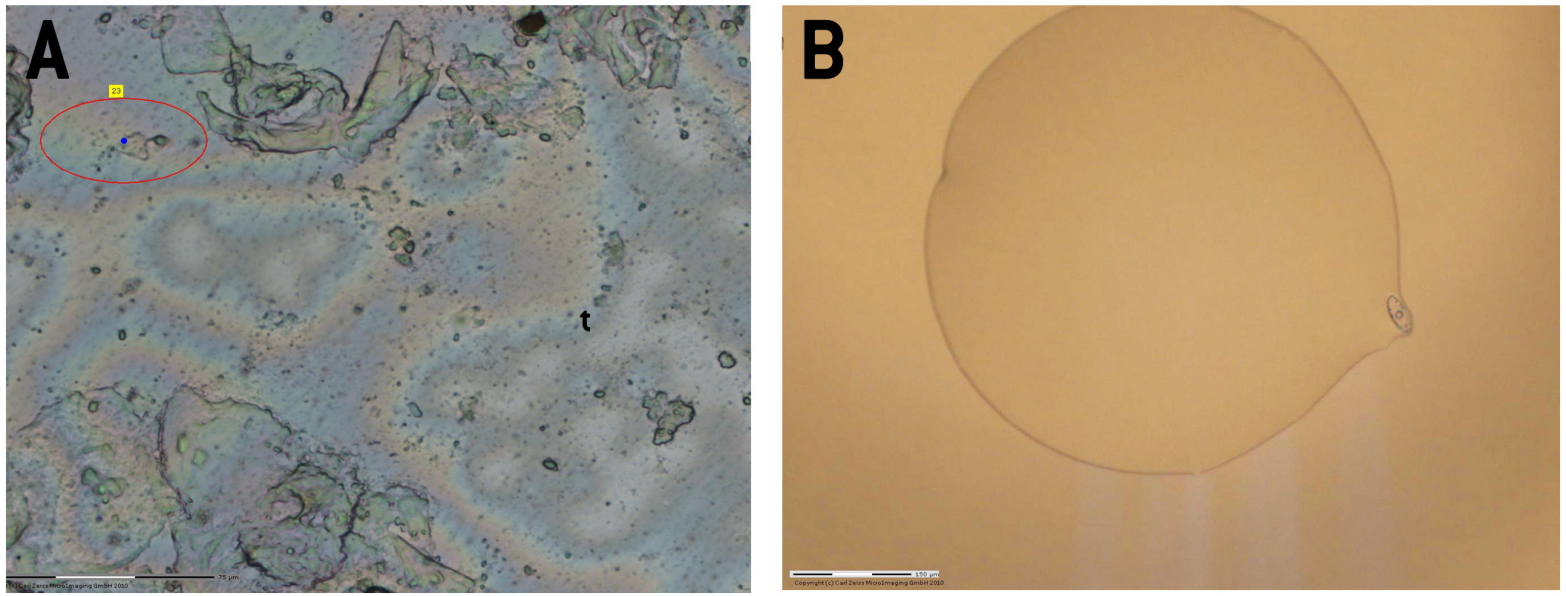
Sperm micrograph (10 × 40). (**A**) One sperm cell was chosen with PALM MicroBeam instrument (Carl Zeiss Ltd) and (**B**) captured and placed on the reaction site.

To assess the single sperm Y-STR analysis method, a volunteer’s sperm sample was examined. Single sperm cell was captured onto one site of the slice using LCM (Figure 1) and genotyped using Y-STR with LV-PCR method. To overcome the random allelic drop-ins, replicate analyses were performed (9). In total, 92 parallel LV-PCRs were performed. Considering that half of the sperm cells contain an X chromosome instead of a Y chromosome, only half of the reactions should yield successful profiles. The actual proportion of Y-STR profiles with more than nine genotyped loci was 48% (44 of 92). Ten complete allelic profiles and 23 acceptable profiles (13-15 loci) were obtained (Table 1). According to Gill’s guidelines (10), an allele can only be confirmed when two or more independent PCR reactions obtain duplicate results. We combined the best five results, which were all complete allelic profiles, to obtain a consecutive genotype (Supplementary Table 1(web extra material 1)) and the consensus genotype was identical with the known profile.

**Table 1:** DNA genotyping results of the single source semen sample

No. of genotyped loci	No. of tests	Call rate (%)
16	10	10.9
13-15	23	25.0
9-12	11	12.0
0-8	48	52.1
Sum of test	92	100

### Casework sample analysis

**Figure 2:**
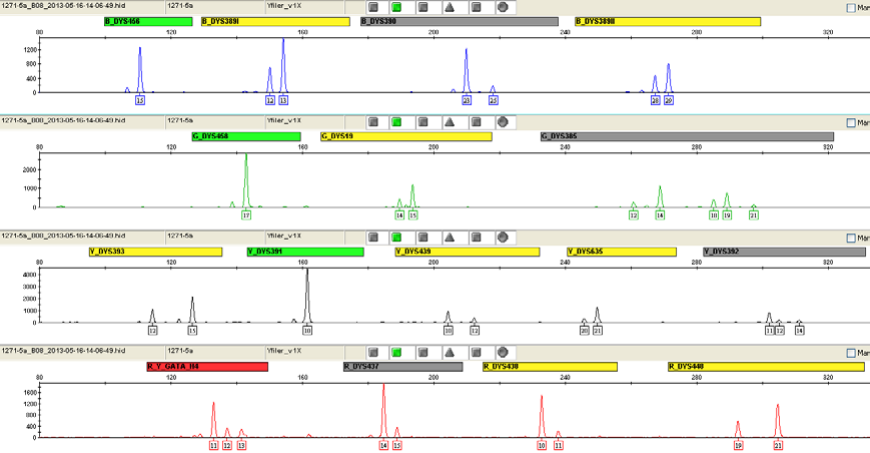
Mixed Y-STR profile of the vaginal swab obtained by the routine method.

**Figure 3:**
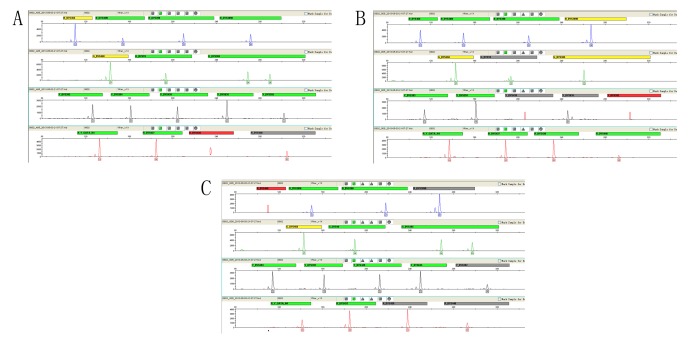
Y-STR electropherograms of the three perpetrators (**A-C**) derived from the casework sample. The red circle indicates allele dropout.

The vaginal swab contained the victim’s epithelial cells and three perpetrators’ sperm cells. Routine preferential lysis DNA analysis yielded a mixed Y-STR profile generated from multiple contributors (Figure 2). Single sperm assay was performed. Ninety-four independent reactions were performed using Yfiler Kit. Two complete allelic profiles and 20 acceptable profiles (13-15 loci) were obtained. Forty-one reactions (44%) produced valid Y-STR profiles of more than nine loci. Twenty-five, ten, and six valid profiles were obtained respectively for the perpetrator A, B, and C (Table 2). According to Gill’s guidelines, we obtained three distinct Y haplotype units (Supplementary Tables 2-4(web extra material 2), Figure 3), which corresponded to three perpetrators’ genotypes. This result served as supportive evidence for the case.

**Table 2:** DNA genotyping results of the 41 reactions that produced valid Y-STR profiles (9-16 loci) in the case sample

No. of genotyped loci	No. of tests for Perpetrator A	No. of tests for Perpetrator B	No. of tests for Perpetrator C	Call rate (%)
16	1	0	1	4.9
13-15	14	3	3	48.8
9-12	10	7	2	46.3
Sum of test	25	10	6	

## Discussion

This study successfully obtained three individual Y-STR profiles in a multi-suspect sexual assault case. Multiple-contributors sperm mixtures in sexual assault cases are challenging to analyze, since they always generate mixed DNA profiles or a profile that only represents the major contributor (11). In the case reported here, the mixed profile was very difficult to interpret as it included three persons’ profiles. Until now, no effective method could distinguish sperm cells originating from different men, although cells containing Y chromosomes (male cells) can easily be distinguished from female cells by labeling the X and Y chromosomes with fluorescence in situ hybridization (FISH) (12). In multi-rapist cases, when valid autosomal STR information cannot be obtained from semen mixtures, Y-STR information can be used to trace the paternal line and implicate a perpetrator (13), and it can also be used to exclude a person that does not match.

In our case testing, 44% of the assays yielded valid profiles (more than nine loci), indicating that we must capture dozens of sperm cells to perform LV-PCR for one Y-STR profile. The consensus profile was used for comparison with the perpetrators’ profile. For the mixed sperm sample, the testing number of the three profiles was different. We assumed that this was mainly due to the different number of sperm cells of the three donors in the semen mixture. The other possible reason may be the random sperm selection process. Our future work will focus on investigating more effective procedures to differentiate the X type sperm and Y type sperm, such as FISH.
