# Supplementary material for: Y-chromosomal haplotyping of single sperm cells isolated from semen mixtures – a successful identification of three perpetrators in a multi-suspect sexual assault case
Source: Croat Med J. 2014 Oct;55(5):537–41. doi: 10.3325/cmj.2014.55.537 (PMC4228287; doi:10.3325/cmj.2014.55.537)
Supplement: Supplementary Table 1 [file CroatMedJ_55_s003.pdf]

Supplementary Table 1. Genotypes of single source semen sample amplified using a Yfiler® kit.

	1	2	3	4	5	Consensus profile	Known profile
DYS456	15	15	15	15	15	15	15
DYS389 I	13	13	13	13	13	13	13
DYS390	25	25	25	25	25	25	25
DYS389 II	31	31	31	31	31	31	31
DYS458	16	16	16	16	16	16	16
DYS19	16	16	16	16	16	16	16
DYS385	11,14	11,14	11,14	11,14	11,14	11,14	11,14
DYS393	13	13	13	13	13	13	13
DYS391	10	10	10	10	10	10	10
DYS439	11	11	11	11	11	11	11
DYS635	23	23	23	23	23	23	23
DYS392	11	11	11	11	11	11	11
Y_GATA_H4	12	12	12	12	12	12	12
DYS437	14	14	14	14	14	14	14
DYS438	11	11	11	11	11	11	11
DYS448	20	20	20	20	20	20	20
