# Supplementary material for: Y-chromosomal haplotyping of single sperm cells isolated from semen mixtures – a successful identification of three perpetrators in a multi-suspect sexual assault case
Source: Croat Med J. 2014 Oct;55(5):537–41. doi: 10.3325/cmj.2014.55.537 (PMC4228287; doi:10.3325/cmj.2014.55.537)
Supplement: Supplementary Tables 2-4 [file CroatMedJ_55_s004.pdf]

Supplementary Table 2. Perpetrator A's Y-STR genotypes.

	1	2	3	4	5	Consensus profile	Known profile
DYS456	15	15	15	-	15	15	15
DYS389 I	13	13	13	13	13	13	13
DYS390	23	23	23	23	23	23	23
DYS389 II	29	29	29	29	29	29	29
DYS458	17	17	17	17	17	17	17
DYS19	15	15	15	15	15	15	15
DYS385	14,19	14,19	14,19	14,19	14,19	14,19	14,19
DYS393	15	15	-	15	15	15	15
DYS391	10	-	10	10	10	10	10
DYS439	10	10	10	10	10	10	10
DYS635	21	21	21	-	21	21	21
DYS392	11	11	11	11	11	11	11
Y_GATA_H4	11	11	11	11	11	11	11
DYS437	14	14	14	14	14	14	14
DYS438	-	-	10	10	10	10	10
DYS448	21	21	21	21	21	21	21

Supplementary Table 3. Results of Perpetrator B's Y-STR genotypes.

	1	2	3	4	5	Consensus profile	Known profile
DYS456	-	15	15	-	15	15	15
DYS389I	12	12	12	12	12	12	12
DYS390	-	23	23	23	-	23	23
DYS389 II	28	-	28	28	-	28	28
DYS458	17	17	17	17	17	17	17
DYS19	-	15	15	15	-	15	15
DYS385	12,18	12,18	12	12	-	12,18	12,18
DYS393	12	12	12	12	-	12	12
DYS391	10	10	10	10	10	10	10
DYS439	12	-	-	12	12	12	12
DYS635	20	-	20	-	20	20	20
DYS392	12	12	-	12	-	12	12
Y_GATA_H4	12	12	12	12	12	12	12
DYS437	15	15	15	15	15	15	15
DYS438	10	-	10	10	10	10	10
DYS448	19	19	19	-	-	19	19

Supplementary Table 4. Results of Perpetrator C's Y-STR genotypes.

	1	2	3	4	5	Consensus profile	Known profile
DYS456	15	-	15	-	15	15	15
DYS389 I	12	12	12	12	12	12	12
DYS390	25	25	25	25	25	25	25
DYS389 II	28	-	28	28	28	28	28
DYS458	17	17	17	17	17	17	17
DYS19	14	14	14	14	13	14	14
DYS385	14	14	14,21	14,21	-	-	14,21
DYS393	12	12	12	12	12	12	12
DYS391	10	-	10	10	10	10	10
DYS439	12	12	12	12	-	12	12
DYS635	-	21	21	21	21	21	21
DYS392	14	14	14	14	-	14	14
Y_GATA_H4	13	13	13	13	13	13	13
DYS437	-	14	14	14	-	14	14
DYS438	11	-	11	11	11	11	11
DYS448	19	-	19	19	19	19	19
